# Effectiveness of Exergaming in Improving Cognitive and Physical Function in People With Mild Cognitive Impairment or Dementia: Systematic Review

**DOI:** 10.2196/16841

**Published:** 2020-06-30

**Authors:** Yinan Zhao, Hui Feng, Xinyin Wu, Yan Du, Xiufen Yang, Mingyue Hu, Hongting Ning, Lulu Liao, Huijing Chen, Yishan Zhao

**Affiliations:** 1 Xiangya School of Nursing Central South University Changsha China; 2 Xiangya-Oceanwide Health Management Research Institute Central South University Changsha China; 3 National Clinical Research Center for Geriatric Disorders Xiangya Hospital Changsha China; 4 Xiangya School of Public Health Central South University Changsha China; 5 School of Nursing University of Texas Health Science Center San Antonio, TX United States; 6 Henan Children's Hospital Zhengzhou China

**Keywords:** mild cognitive impairment, dementia, exergaming, physical, cognitive

## Abstract

**Background:**

Individuals with mild cognitive impairment and dementia have impaired physical and cognitive functions, leading to a reduced quality of life compared with those without such impairment. Exergaming, which is defined as a combination of exercise and gaming, is an innovative, fun, and relatively safe way to exercise in a virtual reality or gaming environment. Therefore, exergaming may help people living with mild cognitive impairment or dementia to overcome obstacles that they may experience regarding regular exercise and activities.

**Objective:**

The aim of this systematic review was to review studies on exergaming interventions administered to elderly individuals with mild cognitive impairment and dementia, and to summarize the results related to physical and cognitive functions such as balance, gait, executive function, and episodic memory.

**Methods:**

We searched Cochrane Central Register of Controlled Trials (CENTRAL), Medline, Embase, PsycINFO, Amed, and Nursing Database for articles published from the inception of the respective databases to January 2019. We included all clinical trials of exergaming interventions in individuals with mild cognitive impairment and dementia for review. The risk of bias was independently evaluated by two reviewers using the Cochrane Collaboration and Risk of Bias in Non-randomized Studies of Interventions tools.

**Results:**

Ten studies involving 702 participants were included for review. There was consistent evidence from 7 studies with a low risk of bias showing statistically significant effects of exergaming on cognitive functioning in people with mild cognitive impairment and dementia. With respect to physical function, 3 of 5 full-scale studies found positive results, and the intensity of most games was classified as moderate.

**Conclusions:**

Overall, exergaming is an innovative tool for improving physical and cognitive function in people with mild cognitive impairment or dementia, although there is high heterogeneity among studies in terms of the duration, frequency, and gaming platform used. The quality of the included articles was moderate to high. More high-quality studies with more accurate outcome indicators are needed for further exploration and validation of the benefits of exergaming for this population.

## Introduction

Mild cognitive impairment is a term used to identify people who are at risk of developing dementia, but the cognitive impairment is so mild that it does not affect daily activities. Symptoms of mild cognitive impairment include memory impairment, language difficulties, attention deficits, disorientation, and altered visuospatial skills [1]. The prevalence of mild cognitive impairment in individuals older than 65 years is approximately 3% to 22% [2-4]. In addition, 5% to 15% of these cases progress to dementia annually, whereas the incidence of mild cognitive impairment in the general population is 1% to 2% per year [5-7].

Dementia is characterized by a group of chronic and progressive symptoms caused by various brain illnesses that affect memory, thinking, behavior, and ability to perform daily activities [8]. Dementia currently affects approximately 50 million people worldwide and is expected to affect 82 million people by 2030 and 152 million by 2050 [9,10]. Dementia is the second leading cause of disability in individuals aged 70 years or older and the seventh leading cause of death worldwide [10,11]. In 2015, the cost of dementia care was estimated at US $818 billion, equivalent to 1.1% of the global gross domestic product, which ranges from 0.2% for low- and middle-income countries to 1.4% for high-income countries. It is estimated that the cost of caring for people with dementia worldwide will increase to US $2 trillion by 2030, which could undermine social and economic development globally and overwhelm health and social services, especially long-term care systems [12].

Owing to the high medical and social burden of mild cognitive impairment and dementia, scientists in various fields have been searching for effective strategies to prevent or delay disease development. In view of the fact that current pharmacological treatments are not only expensive but are also accompanied by significant adverse effects [13], the Food and Drug Administration and experts in leading geriatric organizations recommended that nonpharmacologic approaches be used as the first-line treatment of cognitive impairment [14]. Nonpharmacologic approaches include, but are not limited to, reminiscence therapy [15], reality orientation [16], validation therapy [17], music therapy [18], doll therapy [19], pet therapy [20], and cognitive and physical exercise training [21]. These nonpharmacologic approaches are becoming increasingly preferred by the geriatric population because they have been shown to yield positive results, are easy to use in clinical or home settings, and are inexpensive.

In recent decades, an increasing number of studies have used games for cognitive training in people with mild cognitive impairment or dementia [22-27]. The aim of cognitive training is to maintain or improve specific cognitive functions such as attention, episodic memory, and problem-solving skills using guided training and repetitions of standardized tasks [28]. Game-based interventions are nonpharmacological readily accepted forms for training, and playing games could be an efficient mode to practice mental concentration and memory, making them appropriate for people with cognitive impairment [29-31].

Exergaming, defined as the combination of exercise and gaming, is a relatively new type of intervention in which users must perform physical movements to play games [32]. The design of the games is based on the cognitive enrichment hypothesis, which states that the behaviors of individuals (including cognitive activity, social engagement, exercise, and other behaviors) can influence their level of cognitive function [33]. One idea underlying this hypothesis is to use a rich environment to stimulate brain functioning. This rich environment is reflected when participants play these games, and there is usually a screen displaying information about the game scene. For example, the Kinect sensor incorporates an infrared light and a video camera to create a three-dimensional map in the front area, handheld controllers are also used to manipulate the games [34], and physical movements are captured by the video cameras [35] or weight-sensing platforms [36].

Exergames have been gradually implemented in rehabilitation [37,38], education [39], and other fields [40], and have been widely accepted by a range of populations from children [41,42] to the elderly [43,44]; moreover, positive results were found for individuals with various diseases such as dementia [45], stroke [46], Parkinson disease [34], multiple sclerosis [47], cystic fibrosis [48], and cancer [49]. Recent studies have demonstrated the feasibility, acceptability, and effectiveness of exergaming in improving physical functions such as gait and balance [50], motion control [51], and exercise capacity [52]. Exergaming has been found to be an acceptable method of exercising among older adults [53,54], and is also proven to be safe [55,56], easy to use [53], and enjoyable [53,54].

To the best of our knowledge, there is only one systematic review [57] that has synthesized the existing evidence of exergaming in individuals with dementia. However, due to the limitation of the retrieval strategy, the systematic review included only 3 articles involving a study with an exergaming-integrated training method and two studies using Nintendo Wii training methods. However, few systematic reviews have examined the effectiveness of exergames in improving the physical and cognitive functions in individuals with mild cognitive impairment or dementia. In addition, the latest classification of exergaming includes not only Wii but also handheld controllers, and physical movements captured by video cameras or weight-sensing platforms. Therefore, we conducted a systematic review with a high degree of evidence using the Joanna Briggs Institute methodology for systematic reviews of effectiveness evidence [58]. In reviewing the current literature, we focused on whether an exergaming intervention can indeed be beneficial to the rehabilitation of people with mild cognitive impairment and dementia.

## Methods

### Search Strategy

The search strategy was developed by a researcher who has conducted reviews and a university-level statistics professor. We initially conducted a limited search (with modifications as needed) in the Medline and Embase databases to identify articles relevant to the topic. The text contained in the titles and abstracts of the related articles as well as the index words used to describe the articles were adopted to develop a complete search strategy for the related databases. A combination of search terms was used to identify relevant papers (exergam* or activ* n3 video n3 gam* or activ*) AND (dementia* or alzheimer* or mild cognitive impair* or cognitive impair*), where * represents a wild card allowing the use of other suffixes, and n3 represents the adjacent retrieval operator, which allows three words to be inserted between two words and reverses the order of the words. For more details regarding the search terms, definitions, and variations of input, see Multimedia Appendix 1.

Two authors (Yinan Z and XY) independently identified studies published from the inception of the databases to January 2019. The language was restricted to English by searching the following databases systematically: Cochrane Central Register of Controlled Trials (CENTRAL), Medline, Embase, PsycINFO, Amed, and Nursing Database.

The systematic review is registered with PROSPERO: CRD42019124994. The reporting of the review is consistent with the Preferred Reporting Items of Systematic Reviews and Meta-Analyses (PRISMA) guidelines [59].

### Article Selection

Two reviewers (Yinan Z and XY) independently reviewed the list of potential articles found by the search strategy after removing duplicates with Mendeley reference management software. The inclusion criteria of this review were as follows: (1) randomized controlled trials (RCTs), cluster RCTs, quasiRCTs, and controlled clinical trials; (2) participants were people diagnosed with mild cognitive impairment or dementia; (3) exergaming interventions that combined real-time motions with engaging video games that can help motivate individuals to exercise; (4) comparators included groups who underwent routine exercise, other specific interventions, or no comparative group; and (5) health outcomes reported related to cognitive and physical functions, such as cognitive function, balance and gait, overall physical function, quality of life, behavioralist and neuropsychiatric symptoms, and number of falls. To synthesize more comprehensive evidence, we included both the pilot study and the full-scale study when available.

### Data Extraction

Data were extracted from studies included in the review by two independent reviewers (Yinan Z and XY) using the standardized data extraction tool from the Joanna Briggs Institute Meta-Analysis of Statistics Assessment and Review Instrument [58].

The extracted data included specific details about the author, year of publication, country, study design, populations, study methods, interventions, control group, outcomes, and measurements, along with outcomes of significance to the review objective. Any disagreements that arose between the reviewers were resolved through discussion or consultation with a third reviewer. The authors of the articles were contacted to request missing or additional data when required.

### Study Quality Assessment

Two reviewers independently examined the risk of bias of the included studies using the Cochrane Collaboration tool for assessing the risk of bias [60] (adapted from Higgins and Altman [61]) and Risk of Bias in Non-randomized Studies of Interventions (ROBINS-I) [62]. Any disagreements that arose between the reviewers were resolved through discussion with a third reviewer. Because some of the studies included were pilot studies with small sample sizes, we used Cohen *d* [63] to calculate the effect size.

### Data Analysis

A narrative synthesis was conducted since there were insufficient data available for a statistical meta-analysis. After extracting the required data from relevant journal articles, a descriptive summary was created to summarize the interventions and assess the exergaming interventions used to improve cognitive and physical functions among people with dementia. We calculated Cohen *d* using the Psychometrica program [64]. Because an analysis based on changes from baseline is considered to be more effective and powerful than a comparison based on the final value, for each study, the differences between the baseline and final mean and SD values were included in the analysis; such a baseline change analysis removes a component of interperson variability from the analysis. In articles that did not report SDs, we calculated the SDs from the reported means, along with SEs, 95% CIs, and other relevant information [65].

## Results

### Search Results

**Figure 1:**
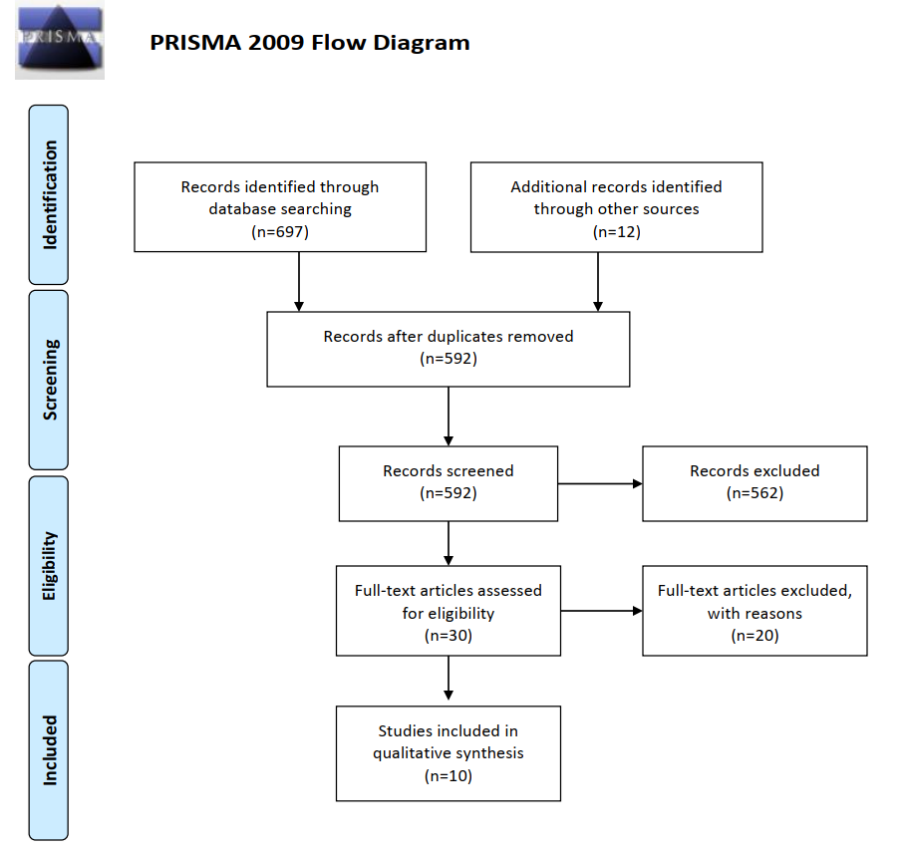
Preferred Reporting Items for Systematic Reviews and Meta-Analyses (PRISMA) flow diagram.

A total of 697 potentially relevant studies were identified in the initial search. After duplicates were removed, the titles and abstracts of 592 records were screened for relevance according to the inclusion criteria, and 30 potentially relevant studies were identified. After viewing the full texts, 20 studies were excluded from the review. In 11 studies, the interventions were multimodal or multicomponent, excluding exergaming. In 7 studies, the participants were elderly but did not have a diagnosis of mild cognitive impairment or dementia. One study was a single-case feasibility study, and another study did not explore the outcome of physical and cognitive function. Finally, 10 articles were included in this study [45,50,52,66-72] (Figure 1).

### Participants

Half of the studies (n=5) were conducted in the United States and the remaining studies were conducted in Germany, Greece, the Netherlands, France, and Pakistan. A total of 702 participants were recruited for these 10 studies, and the data of 597 participants were analyzed after removing duplicates. The mean age of the participants in 9 of the 10 (90%) included studies was 79.8 (SD 7.2) years, and 408 (58.1%) were women. In 8 studies, all participants were diagnosed with mild cognitive impairment or dementia, including mild Alzheimer disease and dementia, whereas the elderly people participating in the other 2 studies included those with and without mild cognitive impairment or dementia. More details on the participants, interventions, comparisons, outcome measures, and results of the included studies are shown in Multimedia Appendix 2.

### Types of Interventions

There were two main categories of exergame interventions implemented in the included studies. The first category corresponded to exergaming training such as balance training, flexible training, and aerobic training, and the second category corresponded to virtual reality-based situational tasks such as riding a bike in a park, crossing roads while avoiding cars, and shopping in a supermarket. The median duration of the intervention was 8 weeks (range 4-24 weeks), although many of the studies used durations ranging from 6 to 8 weeks. Nine articles reported the duration of the sessions, ranging from 30 to 120 minutes, and some studies reported duration ranges, as the completion time was determined by the participants in some cases.

The exergames in 5 studies were implemented on sensor-based platforms [50,67,68,71,72] such as Nintendo Wii-Fit and FitForAll; 4 studies used video camera systems such as Xbox 360 Kinect, X-Torp, and Bike Labyrinth to capture physical movements [52,66,69,70]; and another study used a handheld controller [45]. The interventions were administered by a researcher [45,50,68-70,72], therapist [66], clinical doctor [52], and family caregiver [71]. In the study of Bamidies et al [67], the intervention was carried out in a group setting with psychologists, physical education instructors, researchers, or nurses.

### Outcome Measures

Different indices, including balance, gait, executive function, episodic memory, working memory, emotions, and cognitive performance, were used to evaluate the effects of the exergaming interventions on cognitive and physical functions.

#### Physical Functioning

Physical function was evaluated in 7 full and pilot studies; however, only 3 of these studies showed positive results. Schwent et al [72] used 3 wearable sensors attached to both the lower legs and the lower back of the participants who were instructed to stand for 30 seconds with their feet close together; they were then instructed to stand with their eyes open and closed so that their balance could be assessed, and the authors found a significant result on balance (*P*<.05). Two studies assessed balance using the Berg Balance Scale (BBS), and one showed that the mean BBS score improved to more than 45 points in both groups with the intervention; scores between 41 and 56 points indicate that an individual’s balance function is good, and for elderly people, these scores indicate that they can walk independently. The BBS score improved significantly over time for both groups (*P*<.001); however, there were no significant group-by-time interaction effects on the BBS scores (*P*=.56) [50].

#### Cognitive Functioning

Cognitive function was evaluated in all 10 studies included in the systematic review. Among the 6 full-scale studies, 4 showed positive results. Wiloth et al [45] used a task-specific assessment that included temporal and spatial outcome parameters to measure motor-cognitive performance, and found that exergaming training significantly improved the duration and accuracy parameters (*P*<.001) Bamidis et al [67] used the average *Z*-standardized scores of episodic memories, working memory, and executive function to assess global cognition, and found significantly improved global cognition in the experimental group compared to the control group (*P*=.002). Amjad et al [66] used the Mini-Mental State Examination and Montreal Cognitive Assessment to test participants’ cognitive abilities and found significant interaction effects of the group and time factors on both scores (*P*<.001). They also used the Trail Making Test to assess executive functions, which showed significant improvement (*P*<.001). Another study found that the psychomotor speed of the exergame training group was significantly higher than that of the control group after 12 weeks (*P*=.004) and there was a maintenance effect observed at the 24-week follow-up (*P*=.003); part A of the short form of the Trail Making Test and parts I and II of the abbreviated Stroop Color Word Test were used to assess psychomotor speed. In 4 pilot studies, no positive results were found on cognitive function [69].

### Risk of Bias

**Figure 2:**
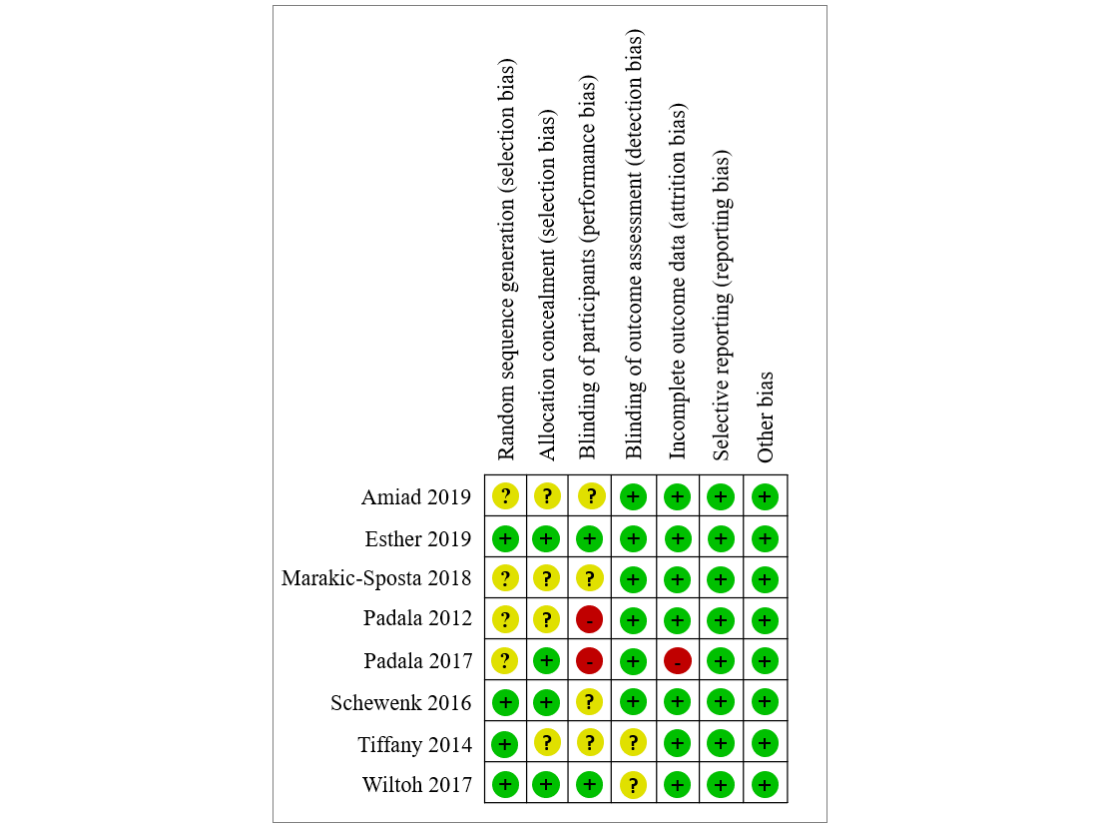
Risk of bias summary.

Eight RCTs and two pretest-posttest studies with a control group design (quasiRCTs) were included in the review. The 8 RCTs were assessed for risk of bias using the Cochrane Collaboration tool [60], which includes the following 7 items: (a) generation of random sequences (selection bias), (b) allocation concealment (selection bias), (c) blinding of the participants and personnel (performance bias), (d) blinding of the outcome assessment (detection bias), (e) incomplete outcome data (attrition bias), (f) selective outcome reporting (reporting bias), and (g) other biases. Two reviewers rated the studies to have “low risk,” “high risk,” or “unclear risk” for each of the categories listed above, corresponding to the green, red, and yellow filled circles, respectively, shown in Figure 2. Moreover, the two quasiRCTs were assessed using the ROBINS-I tool, which has been widely used for evaluating nonrandomized studies. The evaluation results are shown in Table 1.

**Table 1:** Risk of bias assessment according to the ROBINS-I^a^ tool.

Risk of bias	Bamidis et al [67]	Ben-Sadoun et al [52]
Confounding factors	Low	Moderate
Selection of participants	Low	Serious
Classification of intervention	Low	Low
Deviation from intended interventions	Low	Low
Missing data	Low	Low
Measurement of outcomes	Low	Low
Selective reporting	Low	Low
Overall judgment	Low	High

^a^Risk of Bias in Non-randomized Studies of Interventions.

## Discussion

This is the first systematic review that synthesized the existing evidence of exergaming interventions administered to elderly individuals with mild cognitive impairment or dementia and to explore the effect of exergaming interventions on their cognitive and physical functions. The interventions identified in this review differed greatly across studies. The duration and frequency of the interventions also varied greatly; the duration ranged from 4 to 24 weeks, and the total intervention duration ranged from 3 to 36 hours. The frequency ranged from 1 to 5 times a week, and the median was 3, which is consistent with the study of Manera et al [73]. However, after analysis, there was no evidence that longer and more frequent interventions lead to greater improvements in function. Although exergaming is a combination of gaming and exercise, due to the diversity of the platforms used to manage the exergame interventions, it was difficult to determine which exergame was the best for improving cognitive and physical functions. Nevertheless, many studies used sensor-based platforms such as Nintendo Wii. Half of the interventions were carried out in the community, and the rest were administered in hospitals, rehabilitation wards, or nursing homes, indicating that exergaming interventions could be implemented in both environments, but were more common in a community setting. Alzheimer’s Disease International estimated that globally, approximately 84% of elderly patients with dementia currently live in a community [74]. The World Health Organization’s “Rehabilitation 2030” campaign launched in 2017 pointed out that rehabilitation programs should follow a holistic approach for chronic disease management, optimize independence, and prolong community engagement [75]. Because it is inexpensive [49], safe [55,56], and easy to use [53], exergaming can be performed unsupervised even for community-dwelling healthy older adults [76,77]. Exergaming interventions are gradually being used as a physical cognitive rehabilitation tool for elderly people with mild cognitive impairment or dementia living at home in the community.

In a previous literature review, the effectiveness of exergaming training in improving cognitive function was investigated in people with mild cognitive impairment or dementia. The results were consistent with those reported in the study of Karssemeijer et al [69], showing that exergame training significantly improved the psychomotor speed in elderly people with dementia compared with the control group that adopted normal exercise. Moreover, positive results were found in the study of Amjad et al [66] in elderly individuals with mild cognitive impairment, showing improvements in overall cognitive abilities and executive function, and in the study of Bamidis et al [67] in elderly individuals who were healthy or had mild cognitive impairment.

Four of the five formal studies reported that the exergaming intervention improved cognitive function, which is encouraging. In recent years, more and more studies have applied exergames to the cognitive rehabilitation in elderly people with mild cognitive impairment or dementia. The aim of cognitive rehabilitation is to use individualized intervention strategies to address cognitive impairment [78]. Previous studies have shown that an effective approach to treating cognitive impairment requires a highly individualized approach that focuses on the common goals of patients and is interactive [79], and an exergaming intervention meets these criteria. Wiloth et al [45] assigned participants to tasks of different difficulty levels according to their cognitive performance and the intervention was implemented in the presence of the clinicians. In the study of Bamidi et al [67], a group of psychologists, physical education instructors, researchers, and nurses helped each elderly person design a plan that included aerobic, resistance, and strength exercises. However, due to the small sample size, short intervention duration [70-72], and lack of specific exercises [72], a positive result was not obtained in several other preliminary experiments [50,70-72]. In addition, some studies reported “small” to “moderate” effect sizes for the cognitive function results [50,52,72,80].

In the current study, the instrumental activities of daily living and activities of daily living were used to evaluate the independence in performing physical activities of the subjects for three interventions, but only one study showed positive results. However, variations in activities of daily living performance are expected since the extent to which patients with Alzheimer disease lose the ability to perform activities of daily living varies widely [81]. Balance and gait speed are the most widely used physical measurements for evaluating physical function since gait speed [82-84] and functional decline [85] are predictors of survival among elderly people. Four studies included balance in the outcome measures, three of which found positive results and the other found a moderate effect size in physical function among participants with neurodegenerative diseases. These results indicate that exergames do improve postural control in older adults compared to walking training [50,71,72,85]. This conclusion can be supported by the theory of planned behavior, which postulates that subjective norms and behavioral attitudes determine individual behavioral intentions and the latter determines individual behaviors [86]. An interesting result was found with respect to use of the same exergaming intervention (Nintendo Wii-Fit) and control intervention (walking program), both of which lasted for 8 weeks. One study showed no significant group-by-time interaction effects on the BBS scores (*P=.*56) [50], but the other showed that among the participants who completed the test, there was a significant group-by-time interaction effect on the primary outcome measure, the BBS score (*P*<.05) [71], which we consider to be a very promising result. This discrepancy may have been partly caused by a small sample size or low test efficiency. Additional studies with sufficient sample sizes and high quality should be conducted to verify the results.

According to the literature review, in addition to cognitive and physical functions, we also found that exergames affect other outcomes. Apathy is a common neuropsychiatric syndrome observed across many neurocognitive and psychiatric disorders. In the study of Valeria et al [87], 20 experts reported that information and communications technology is “very appropriate” for apathy nonpharmacological treatment. Among all the studies included in this review, none of the studies using measurement tools detected changes in apathy before and after the intervention; however, we found some benefits in terms of motivation and compliance.

Motivation or compliance was evaluated or explored in 70% of the studies reviewed. All 7 studies that evaluated motivation or compliance agreed that the exergames increased or enhanced the participants’ motivation to engage in rehabilitation activities. Although exercise therapy has been recommended to improve the cognitive and physical functions in people with dementia or mild cognitive impairment in recent years [88], there are still many barriers to exercise, such as lack of motivation and limited access to exercise facilities [89]. Exergames provide sensory feedback through auditory, visual, and tactile stimulation [90], and can further maintain the motivation of individuals [66]. Therefore, exergaming interventions have a high adherence rate among rehabilitation methods. Ben-Sadoun and colleagues [52] found that both groups in the study experienced only positive emotions, which was consistent with previous findings on exergames in subjects with mild cognitive impairment and Alzheimer disease. In the study of Hughes et al [68], the majority of participants were “very much” satisfied with the intervention. An open-label RCT among elderly people with mild cognitive impairment showed that exergaming interventions can reduce individuals’ fear of falling compared with no training [72].

Despite the positive impact, some limitations of our study must be considered when interpreting the results. The studies included in this review varied substantially in terms of the consoles used, games played, participants’ stages of mild cognitive impairment or dementia, and the outcomes assessed, making analyses of the effects of exergaming difficult, and a meta-analysis could not be performed. To gather comprehensive evidence, we included pilot studies with small sample sizes. Therefore, it is difficult to draw firm conclusions from the results of the analyses due to the limited statistical power.

This is the first systematic review that assessed the effectiveness of exergaming interventions in improving the cognitive and physical functions of elderly people with mild cognitive impairment or dementia. The studies included in the analysis were heterogenous in terms of the modalities used to administer the interventions, health outcome evaluations performed, and outcomes assessed. Overall, exergame interventions can improve cognitive and physical function to some extent, and more high-quality studies with more accurate outcome indicators are needed for further exploration.

## Appendix

Multimedia Appendix 1 Search strategy.

Multimedia Appendix 2 Study characteristics.

## Abbreviations

BBS: Berg Balance Scale
CENTRAL: Cochrane Central Register of Controlled Trials
PRISMA: Preferred Reporting Items of Systematic Reviews and Meta-Analyses
RCT: randomized controlled trial
ROBINS-I: Risk of Bias in Non-randomized Studies of Interventions
