# Supplementary material for: Effectiveness of Exergaming in Improving Cognitive and Physical Function in People With Mild Cognitive Impairment or Dementia: Systematic Review
Source: JMIR Serious Games. 2020 Jun 30;8(2):e16841. doi: 10.2196/16841 (PMC7367532; doi:10.2196/16841)
Supplement: Multimedia Appendix 1 [file games_v8i2e16841_app1.docx]

**Multimedia appendix 1- search strategy**

We initially conducted a limited search (with changes as needed) for MEDLINE and EMBASE to determine articles on this topic. The text words contained in the title and summary of the related article, as well as the index words used to describe the article, are used to develop a complete search strategy for the name of the reported related database.

We will search terms: (exergam* or activ* n3 video n3 gam* or activ* n3 videogam* or human n3 computer n3 interaction or virtual n3 reality or virtual n3 world or augment* n3 reality or mobile n3 app* or exert* n3 interfac* or electronic n3 gam* or activ* n3 gam* n3 play or virtual n3 rehab* or augment* n3 rehab* or mHealth or Wii or Eye n3 Toy or Eyetoy or Kinect or DDR or Dance n3 Dance n3 Revolution or IREX) and (dementia* or alzheimer* or cognitive impairment or neurodegenerative disorder or neuro cognitive disorder)

|  |
| --- |

|  |
| --- |
