# Supplementary material for: Effectiveness of Exergaming in Improving Cognitive and Physical Function in People With Mild Cognitive Impairment or Dementia: Systematic Review
Source: JMIR Serious Games. 2020 Jun 30;8(2):e16841. doi: 10.2196/16841 (PMC7367532; doi:10.2196/16841)
Supplement: Multimedia Appendix 2 [file games_v8i2e16841_app2.pdf]

Multimedia Appendix II-Characteristics of included studies.

Study	Design	Population:n enrolled(IG/CG), n completed (IG/CG), location	Disease	Setting	Delivered by	Exergaming intervention	Control/comparison	Outcomes & measurement	Finding
Wiloth et al/ 2017	Double-blinded randomized controlled intervention trail	99(56/43), 99(56/43), Germany	Dementia	Rehabilitation wards, nursing homes and community	Research staff	IG underwent a exergaming training program for 10 weeks (1.5h, twice a week) through **Physiomat**.<br><br>**Physiomat** games include that FTBT and PTMT. In FTBT, the patient moves the yellow ball on screen as fast as possible while holding onto the handles. In PTMT, patients are asked to move the cursor on the screen to connect the digits as fast as possible.	CG underwent a placebo group training for 10 weeks (1h, twice a week).	①Motor-cognitive Physiomat performance: derived from the data stream during the game task, including temporal and spatial.<br>②The number of successfully performed tasks: PTMT score.	Significantly improved the duration, accuracy and PTMT score.
Tiffany et al/ 2014	Randomized controlled trail	20(10/10), 19(10/9), USA	MCI	Community	Research staff	IGs met for 24weeks (1.5h, once per week) through **Nintendo Wii**.<br><br>Wii games includes Wii Sports (bowing, golf, tennis, and baseball), Boom Blox, Wii Play, Sports Resort. In weeks 10 and 20, the groups competed in a "Wii tournament"	Health aging education program.<br><br>CGs met to learn about and discuss age-specific health-related topics with professionals, accepted handouts to read. Also, they join the group activities and competed in a Jeopardy style	①Cognitive performance: CAMCI, Cognitive Self-Reported Questionaire-25.<br>②Physical function: gait speed (6MiWT)<br>③Performance-based instrumental activities of daily living (The Timed Instrumental Activities of Daily Living),	High satisfaction.
Bamidis et al/2015	Pre-post-test design	322(237/85), 229(163/66), Greece	Ranging from cognitively healthy individuals to individuals with mild cognitive impairment or dementia	Day care centers, hospitals, senior care centers, a memory outpatient center, local parishes, at university campus facilities, and at participant's homes	Group setting with psychologists, physical educator, researchers, or nurses	IGs accepted **physical and cognitive training** for 7-8weeks of 1h FitForAll exergames (10min warming up, 4x10-15min, 5min cooling down)<br><br>**Physical training** includes aerobics (Hiking&Cycling), strength (weightlifting&resista nce training gradually revealing pictures), balance (Ski Jump, Arkanoid, Apple tree, Fishing, Golf) and flexible training (stretching and warm- up training)<br>**Cognitve training:** six tasks targeting auditory processing and working memory.	Passive control group	①Cognitive outcomes: Greek versions of the California Verbal Learning Test, the Digit Span Test, the Trail Making Test.<br>②Specific cognitive functions and global cognition: calculated by averaging z-standardized sub-scores of the three cognitive tests.<br>③Psychological, Physical, and Daily Living Outcomes: quality of life (WHOQOL-BREF), depression(GDS-short), physical fitness(SFT), Daily life functioning(IADL)	Significantly improving in global cognition.

Schwenk et al/2016	Open-label randomized controlled trial	22(11/11) , 20(11/9), USA	MCI	Community	Research staff	IG underwent balance training for 4 weeks (45min, twice a week)<br><br>Training include: ankle point-to-point reaching tasks and virtual obstacle-crossing tasks.	CG receive no training.	①User experience: A questionnaire with 10 Likert-scale questions.<br>②physical function: Balance, gait<br>③depression: CES-D<br>④Fear of Falling: Short Falls Efficacy Scale International (FES-I).<br>⑤Cognitive performance: Montreal Cognitive Assessment.	Significantly improving in balance and reducing in fear of falling.
Amjad et al/2019	Randomized controlled trail	44(22/22), 38(18/20), Pakistan	MCI	Rehabilitation department	Therapist	IG receive the Cognitive training with Xbox 360 Kinect game for 6 weeks (30min, 5 days a week)<br><br>Training include 5 domains: logic, physical, memory, reflexes and math.	CG perform normal joint range of motion and stretching exercises of upper and lower limbs for 6 weeks (30 mins, 5 days a week)	①Cognitive abilities: mini-mental state examination (MMSE) and Montreal cognitive assessment scale (MoCA).<br>②Executive functions: trail making test (TMT).	Significantly improving in cognitive abilities and executive function.
Karssemeijer et al/2019	Randomized controlled trail	115(38/39/38) , 115(38/39/38) , Netherlands.	Dementia	Community	Research staff	IG receive three training sessions per week for 12 weeks.<br><br>**Exergaming training** consisted of a combined cognitive-aerobic bicycle training.<br><br>**Single aerobic exercise** consisted of cycling on a stationary bike that was not connected to a video	CG receive training that consisted of relaxation and flexibility exercises.	①Objective executive functioning: four neuropsychological tasks.<br>②Cognitive function: episodic memory (Location Learning Test—Revised), working memory (WAIS-III Digit Span and WMS-III Spatial Span), and psychomotor speed (short form of Trail Making Test Part A and the abbreviated Stroop Color Word Test parts I and II).	Significantly improving in psychomotor speed.
Ben-Sadoun et al/2015	Case controlled study	18(10/8),17(10/7), France.	Cognitive impairment and healthy elderly	Hospital	Clinical doctor	IG follow a 13 training with 13-session training with X-Torp over 5 weeks, for a total of 10 hours of game stimulation.<br><br>The exergaming is a sea simulation game that uses a scenario mode to do sea work for the participants' hand and leg.	CG obtain the same intervention measures as the intervention group	①Physical fitness: balance (SPPB), gait(10MeWT), sit to stand transfer (TUG), aerobic exercise capacity (6MiWT).<br>②Cognitive functions: MMSE, FAB, SCB Memory and SCB Fluency, TMT, DSST and DMS48.	Participates experience positive emotions and reported a 'moderate' to 'high' perceived competence.
Padala et al/2012	A prospective randomized pilot study	22(11/11), 22(11/11), USA	Mild AD	An assisted living facility	Research staff	Wii-Fit for 8 weeks (30min, 5 times a week)<br><br>Exergaming include: strength training (single leg extensions, lunges, and torso twists), yoga (half- moon, warrior pose, chair and salutation) and balance games (soccer heading, ski slalom, ski jump, table tilt, balance bubble, and penguin	CG obtain the walking program indoors for 8 weeks (30min daily, 5 times per week)	①Physical function: balance (BBS, TT, TUG), functional ability (ADL, IADL)<br>②Cognitive function: MMSE<br>③Quality of life: (QOL-AD)	Significant improvement on BBS and TT.

Padala et al/2017	A prospective randomized controlled parallel- group trial	30(15/15), 30(15/15), USA	history of mild AD & MMSE score ≥ 18.	Community	Family caregiver	Wii-Fit for 8 weeks (30min, 5 times a week)<br><br>Exergaming include: balance, aerobic, strength training and yoga.	CG obtain the walking program either indoors or outdoors, 30 min daily, 5 times per week, for 8 weeks. Walking at own pace.	①Physical function: balance (BBS), functional ability (ADL, IADL)<br>②Cognitive function: 3MS<br>③Quality of life: QOL-AD<br>④Fear of falling: ABC and FES	Significant improvement on BBS, ABC and FES.
Mrakic-Sposta et al/2018	A prospective randomized pilot study	10(5/5), 8(4/4), USA	Mild-moderate cognitive impairment	Community	Research staff	IG perform the physical and training for 6 weeks (40-45min, 3 sessions per week)<br><br>The VR-based training session include three environments: riding a bike in a park, cross the road-avoiding cares and shopping in the supermarket.	Blank control group	①Cognitive function: entire neuropsychological battery (MMSE), episodic verbal memory (RAVLT_I and RAVLT_D), Visuo-spatial functions (ROCFT)<br>②Physical functions: executive functions (FAB), executive functions (VF), independency in daily life (FAQ)<br>③Oxidative stressors Determination, Antioxidant Capacity, Enzymatic Assays.	Significant decrease in OxS. A greater improvement in the executive test, memory functions and verbal fluency but no statistical significance.

Notes: IG, intervention group; CG, control group; FTBT, Physiomat-Follow-The-Ball Task; PTMTs, Physiomat-Trail-Making Tasks; CAMCI ,Computerized Assessment of Mild Cognitive Impairment; WHOQOL-BREF, short version of the World Health Organization Quality of Life questionnaire; GDS, Geriatric Depression Scale; SFT, Senior Fitness Test; IADL, Instrumental Activities of Daily Living Scale; CES-D, Center for Epidemiologic Studies Depression Scale; FES-I, Falls Efficacy Scale International; MMSE, Mini Mental State Exam; MoCA, Montreal cognitive assessment scale, TMT, trail making test; WAIS-III, Wechsler Adult Intelligence Scale 3rd edition; WAIS-III, Wechsler Adult Intelligence Scale 3rd edition; SPPB, Short Physical Performance Battery ; 10MeWT, the 10-Meter Walk Test; TUG, the Time Up and Go; 6MiWT, 6 Minutes Walking Test, FAB, Frontal Assessment Battery; SCB memory, Short Cognitive Battery memory; DSST, Digit Symbol Substitution Test; DMS 48, Delayed Matching to Sample 48 explicit working memory; TT, Tinetti Test; BBS, Berg Balance Scale; ADL, activities of daily living; QOL-AD, quality of life-Alzheimer's disease; 3MS, Modified Mini Mental; ABC, Activities Specific Balance Scale; FES, Falls Efficacy Scale; RAVLT_I and RAVLT_D, Immediate Recall and Delayed of Rey Auditory; FAQ, Functional Activity Questionnaire; OxS, oxidative stress.
